# The Absence of Tryptase Mcpt6 Causes Elevated Cellular Stress in Response to Modulation of the Histone Acetylation Status in Mast Cells

**DOI:** 10.3390/cells8101190

**Published:** 2019-10-02

**Authors:** Sebastin Santosh Martin, Fabio Rabelo Melo, Gunnar Pejler

**Affiliations:** 1 Department of Medical Biochemistry and Microbiology, Uppsala University, BMC, Box 582, 75123 Uppsala, Sweden; 2 Department of Anatomy, Physiology and Biochemistry, Swedish University of Agricultural Sciences, 75007 Uppsala, Sweden

**Keywords:** mast cells, tryptase, cell stress, Psme4, glutathione, thioredoxin

## Abstract

Mast cells contain large amounts of proteases stored within their secretory granules. Previously we showed that one of these proteases, tryptase, in addition to its location within granules, can also be found within the mast cell nucleus, where it has the capacity to affect the acetylation profile of nucleosomal core histones in aging cells. Based on this notion, and on the known sensitivity of mast cells to modulation of histone acetylation, we here asked whether tryptase could impact on the responses against cellular stress caused by disturbed histone acetylation status. To address this, wild-type and tryptase-deficient (Mcpt6^−/−^) mast cells were subjected to cell stress caused by trichostatin A (TSA), a histone deacetylase inhibitor. Wild-type and Mcpt6^−/−^ mast cells were equally sensitive to TSA at an early stage of culture (~8 weeks). However, in aging mast cells (>50 weeks), tryptase-deficiency led to increased sensitivity to cell death. To address the underlying mechanism, we assessed effects of tryptase deficiency on the expression of markers for proliferation and cell stress. These analyses revealed aberrant regulation of thioredoxin, thioredoxin reductase, glutaredoxin, and glutathione reductase, as well as blunted upregulation of ribonucleotide reductase subunit R2 in response to TSA in aging cells. Moreover, the absence of tryptase led to increased expression of Psme4/PA200, a proteasome variant involved in the processing of acetylated core histones. Altogether, this study identifies a novel role for tryptase in regulating the manifestations of cell stress in aging mast cells.

## 1. Introduction

Mast cells (MCs) are immune cells having a profound impact on allergic conditions [1]. In addition, they can influence a large variety of other pathological conditions, such as pruritus, cancer, various autoimmune disorders, chronic inflammation, urticaria, neurofibromatosis, transplantation, wound healing, systemic lupus erythematosis, obesity, and fibrosis [2,3,4,5,6,7,8,9,10]. In most of these settings, MCs are considered as being detrimental. However, beneficial functions of MCs have also been described, in particular in the defense against various toxins and against bacterial, viral, and parasite infections [11,12,13,14,15,16].

A hallmark feature of MCs is their remarkably high content of secretory granules. These are filled with a plethora of preformed inflammatory mediators, including histamine and other bioactive amines, cytokines, growth factors, lysosomal hydrolases serglycin proteoglycans, and a panel of MC-restricted proteases. The latter include chymases, tryptases, and carboxypeptidase A3 (CPA3) [17], of which tryptases and chymases are serine endo-proteases whereas CPA3 is a metallo-exopeptidase [18,19,20]. When MCs are activated, which can be accomplished by a variety of triggers, the preformed secretory granule compounds are released [21]. MC activation will also lead to the *de novo* production of additional compounds. These include various lipid-derived mediators such as platelet activating factor, prostaglandins, and leukotrienes. In addition, MC activation can lead to *de novo* synthesis of numerous cytokines and growth factors, including IL-6, IL-4, TNF, vascular endothelial growth factor, and many others [21,22,23,24]. Altogether, MC activation can thus result in the release of an impressing array of pro-inflammatory compounds, both from preformed stores and after *de novo* synthesis, and the combined effects of these can give rise to powerful inflammatory responses.

When assessing the function of MC tryptase we previously found intriguing evidence that, in addition to its location within the MC secretory granules, tryptase could also be found within the nucleus [25]. Moreover, we noted that tryptase has the ability to cause N-terminal truncation of nucleosomal core histones [25]. It is now well established that the N-terminal ends of nucleosomal core histones are important targets for epigenetic modification, including acetylation, methylation, and phosphorylation [26,27], and our previous findings revealed that the absence of tryptase resulted in an altered core histone acetylation profile in MCs [28]. Notably, the effects of tryptase on histone acetylation were predominantly seen after long-term culture of MCs, suggesting that the effects of tryptase on histone modification are age-dependent [28].

In another recent report it was demonstrated that MCs, as manifested in mastocytosis, are remarkably sensitive to apoptosis induced by histone deacetylase (HDAC) inhibition [29]. Hence, these studies have established that tryptase has the ability to regulate the histone acetylation landscape of MCs and that MCs are remarkably sensitive to cell stress caused by alterations of the histone acetylation status. Based on these notions together we here hypothesized that tryptase can have an impact on how MCs respond to cell stress triggered by modulation of the histone acetylation profile. Indeed, we demonstrate that the absence of tryptase results in increased sensitivity to cell stress downstream of HDAC inhibition, and that this effect is dependent on the age of the MCs.

## 2. Materials and Methods

### 2.1. Reagents

ActinRed^TM^ 555, ActinGreen^TM^ 488, NucBlue Hoechst 33342 were from Molecular Probes (Oregon, OR, USA). AnnexinV-FITC was from BD bioscience (San Jose, CA, USA). DRAQ7^TM^ was from Biostatus (Shepshed, UK). Trichostatin A (TSA) was from Sigma-Aldrich (Steinheim, Germany). May-Grünwald Eosine-methylene blue solution (product number: HX68862424) and Giemsa Azur-Eosine-methylene blue solution (product number: HX128350) were from Merck KGaA (Darmstadt, Germany). SYBR GreenER SuperMix and Rox reference dye were from Invitrogen (Carlsbad, CA, USA).

### 2.2. Bone Marrow-Derived MCs

Femurs and tibiae from mice of the same gender and age were recovered, and MCs were obtained by culturing bone marrow cells in Dulbecco’s Modified Eagle’s medium (DMEM) (SVA, Uppsala, Sweden), supplemented with 30% WEHI-3B conditioned medium, 10% heat-inactivated fetal bovine serum (FBS) (Invitrogen), 50 μg/mL streptomycin sulfate, 60 μg/mL penicillin G, 2 mM L-glutamine (SVA), and 10 ng/mL mouse recombinant IL-3. The cells were kept at 0.5 × 10^6^ cells/mL, at 37 °C in 5% CO_2_; the medium was changed once a week [30]. The animal experiments were approved by the local ethical committee (Uppsala Animal Ethics Committee; Dnr 5.8.18-05357/2018).

### 2.3. May-Grünwald/Giemsa Staining

To prepare cytospin slides, 100 μL of cell suspensions were centrifuged onto the slides for 5 min at 500 rpm. The slides were air-dried and incubated with 100% May-Grünwald Eosine-methylene blue solution for 5 min and then with 50% May-Grünwald Eosine-methylene blue solution for 1 min, followed by 15 min incubation in 2.5% Giemsa Azur Eosin-methylene solution and washing in H_2_O. The slides were dried before mounting. Experiments were repeated with three different batches of cells.

### 2.4. Cell Viability

Cells were washed and resuspended in Annexin V binding buffer (BD Biosciences, Franklin Lakes, NJ, USA) and stained with Annexin V (BD Biosciences) and DRAQ7™ (Biostatus Ltd., Shepshed, UK). Subsequently, stained cells were analyzed with an Accuri flow cytometer (BD Biosciences) for assessment of cell death. Data analysis was performed using the FlowJo software (TreeStar Inc., Ashland, OR, USA).

### 2.5. Quantitative Real-Time PCR

NucleoSpin^®^ RNA isolation kit from (MACHEREY-NAGEL GmbH & Co. KG) was used for total RNA isolation. RNA purity and concentrations were assessed with a Nanodrop device. Amounts of 200 ng of highly purified RNA (A260/280 > 1.95) were used for cDNA synthesis (Bio-Rad, Solna, Sweden). SYBR GreenER SuperMix was used along with primer mix and cDNA for the quantitative real-time PCR (qPCR) with a 7900HT Fast Real-Time PCR System (Thermo Fisher Scientific). The following primers were used: Gapdh forward: 5′CTC CCA CTC TTC CAC CTT CG-3, Gapdh reverse: 3′CCA CCA CCC TGT TGC TGT AG-5′; Trx1 Forward -5′CAT GCC GAC CTT CCA GTT TTA-3; Trx1 Reverse-3′TTT CCT TGT TAG CAC CGG AGA-5′; (Thioredoxin reductase) Txnrd1 Forward -5′ GGG TCC TAT GAC TTC GAC CTG-3; Txnrd1 Reverse -3′AGT CGG TGT GAC AAA ATC CAA G-5′; Grx1 Forward -5′AGG TGG TCG TGT TCA TCA AGC-3′; Grx1 Reverse -3′AGA AGA CCT TGT TTG AAA GGC AG-5′; (Glutathione reductase) Gsr Forward -5′GAC ACC TCT TCC TTC GAC TAC C-3′; Gsr Reverse -3′CAC ATC CAA CAT TCA CGC AAG-5′; (Ribonucleotide reductase R1) Srrm1 Forward -5′TCT CGG TCA CGA TCC AAA TCT-3′; Srrm1 Reverse -3′CTA TGT CTC CGT CTT GGT CTA GT-5′; (Ribonucleotide reductase R2) Rrm2 - Forward -5′GCC GAG CTG GAA AGT AAA GC-3′; Rrm2 - Reverse -3′TCA TGG TAC TCG ATG GGA AAG A-5′; (Ribonucleotide reductase p53R2) Rrm2b- Forward -5′AGA GCA AAC GCA AAT CAA AGG A-3′; Rrm2b -Reverse -3′CGA GAC CGA CTA CGA GAA GTA TC-5′; psme4- Forward -5′AGC GTC AAC AAG ATA AGA ATG CT-3′; psme4-Reverse -3′GCC CGA TTC CTA TAT GCT CAA A-5′.

### 2.6. Confocal Microscopy

Cytospin slides were prepared from aliquots (100 μL) of cell suspensions and cells were then fixed with 4% paraformaldehyde in PBS for 15 min. An amount of 100 μL of 50 μg/mL digitonin solution in PBS was added to each glass slide and incubated for 10 min at room temperature. Next, 100 μL anti-Psme4/PA200 antibody (1:500) in TBS/1% BSA and/or isotype control at the same concentration were added and left overnight at 4 °C, followed by washing 3 times with TBS-T. Next, Alexa-conjugated secondary antibody diluted (1:1000) in TBS/1% BSA was added and incubated for 1h at room temperature. ActinRed/Green^TM^ (Molecular Probes) probes were used to stain cells. The slides were kept in the dark, and washed 3 times with TBS-T between each step. Finally, 100 μL of Nucblue^TM^ (Molecular Probes) probes in TBS/1% BSA was added for 20 min, followed by 3 times washing with TBS-T. The slides were mounted with SlowFade^®^ gold antifade mounting medium (Life Technologies). Samples were analyzed using a laser-scanning microscope equipped with ZEN 2009 software (LSM 710; Carl Zeiss, Berlin, Germany).

### 2.7. Flow Cytometry

MCs (0.5 × 10^6^ cells/mL) were fixed and permeabilized using eBioscience^TM^ intracellular fixation and permeabilization buffer set (eBioscience, San Diego, CA, USA). Subsequently, cells were incubated with rabbit anti-Psme4/PA200 antibody (1:250) and/or isotype control at the same concentration for 1h at room temperature. Cells were then washed and incubated with anti-rabbit Alexa488 antibody (1:1000) for 30 min at room temperature. After washing twice, cells were resuspended to 500 μL in PBS and assessed by flow cytometry (BD Accuri C6 plus, BD bioscience, San Jose, CA, USA). Data analysis was performed using the FlowJo software (TreeStar Inc., Ashland, OR, USA).

### 2.8. Statistical Analysis

Data were analyzed by either Student’s t-test, Sidaks multiple comparison test or by Two-way ANOVA using GraphPad Prism 7 software (La Jolla, CA) and are presented as mean values ± SEM; * *p* < 0.05, ** *p* < 0.01, *** *p* < 0.001, **** *p* < 0.0001. Shown results are representative of ≥2 individual experiments (*n* ≥ 3).

## 3. Results

**Figure 1:**
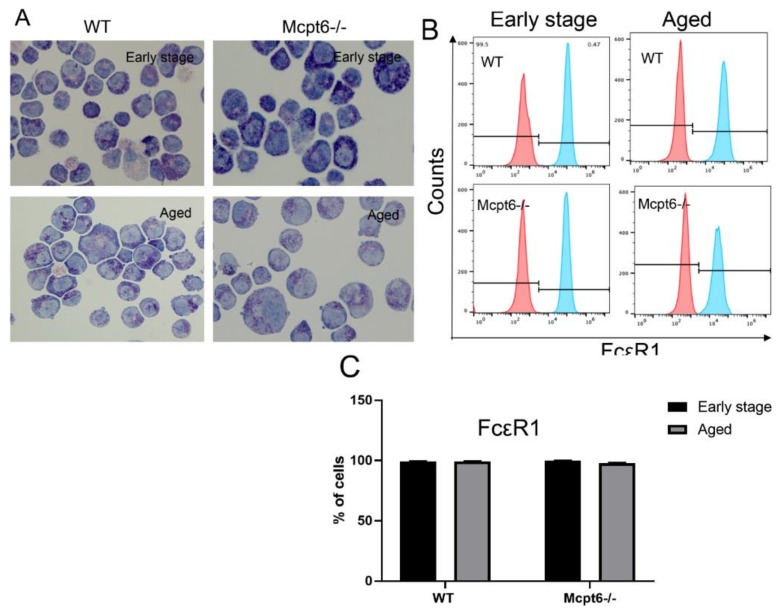
Early stage and aged wild-type (WT) and tryptase-deficient (Mcpt6^−/−^) mast cells (MCs) have similar morphology and express the high affinity IgE receptor. (**A**) Early stage (~8 weeks) and aged (>50 weeks) WT and Mcpt6^−/−^ bone marrow-derived MCs were stained with May-Grünwald/Giemsa. Images representative of three independent cultures are shown. Note that WT and Mcpt6^−/−^ MCs display similar content of May-Grünwald/Giemsa-positive granules (**B**) Histogram showing FcεR1 staining of early stage and aged WT and Mcpt6^−/−^ MCs, analyzed by flow cytometry. (**C**) Quantification of FcεR1^+^ cells (*n* = 3; representative of three independent experiments), showing that WT and Mcpt6^−/−^ MCs express equal levels of FcεR1. Data are presented as mean values ± SEM and Sidaks multiple comparison test or by Two-way ANOVA.

To study the impact of HDAC inhibition on MCs, and if such effects are dependent on tryptase, we developed bone marrow-derived MCs from wild-type (WT) and tryptase-deficient (Mcpt6^−/−^) mice. Cells were cultured for extended time periods, to allow an assessment of age-dependent effects of HDAC inhibition on MCs. Morphological assessments showed that both the WT and Mcpt6^−/−^ MCs were, as expected, densely granulated (Figure 1A). Moreover, the extent of granulation was similar in the early stage and aged cells (Figure 1A). However, it was noted that Mcpt6^−/−^ cells were somewhat larger than corresponding WT cells (Figure 1A). Importantly, almost 100% of the cells, both from WT and Mcpt6^−/−^ mice were positive for the high affinity IgE receptor (FcεRI), confirming that they represent differentiated MCs (Figure 1B,C).

**Figure 2:**
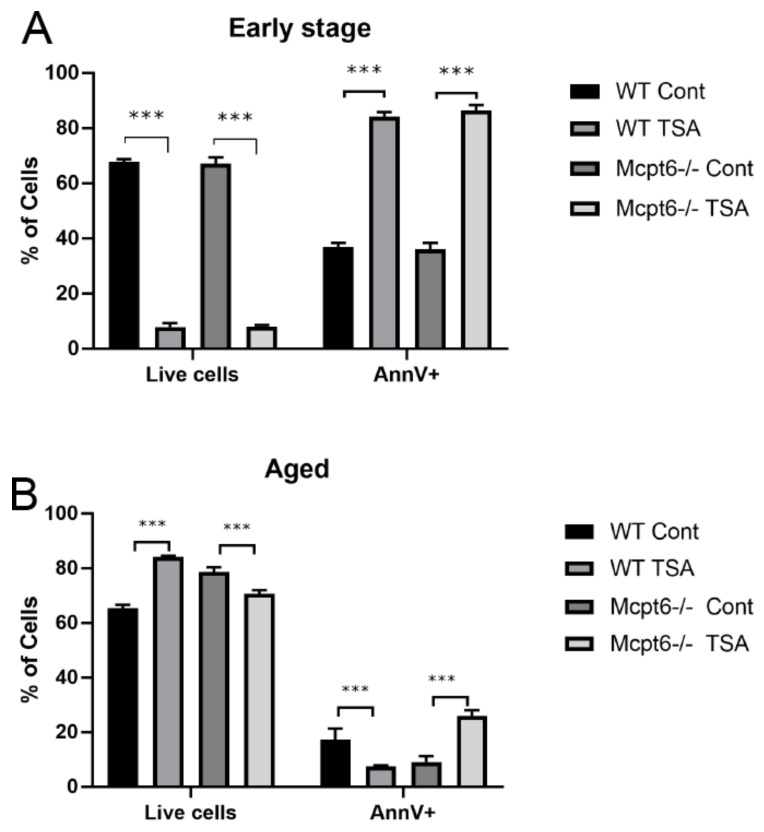
The absence of tryptase Mcpt6 affects the sensitivity to trichostatin A (TSA) in aged MCs. Early stage (**A**) and aged (**B**) MCs were treated with non-toxic concentrations of Trichostatin A (TSA) for extended periods of time (14 days), with repeated addition of TSA on every 4th day. The cells were stained and analyzed using flow cytometry for apoptotic markers. Note that the early stage WT and Mcpt6^−/−^ MCs were equally sensitive to TSA, whereas only Mcpt6^−/−^ aged cells showed reduced viability in response to TSA. Data are presented as mean values ± SEM, pooled from three different experiments, analyzed with Sidaks multiple comparison test or by Two-way ANOVA. **** *p* ≤ 0.0001, ** *p* < 0.005. Cont, vehicle control.

In early stage cultures (~8 weeks), both WT and Mcpt6^−/−^ MCs were highly sensitive to long-term treatment with HDAC inhibitor (Trichostatin A; TSA). As seen in Figure 2A, TSA caused a reduction in the number of viable cells, with approximately equal effects seen in cultures of WT and Mcpt6^−/−^ cells. In agreement with this, TSA caused an increase in the proportion of dead cells as measured by Annexin V positivity (AnnV^+^), with similar effects seen on WT and Mcpt6^−/−^ cells (Figure 2A). In contrast, the effects of TSA were different on the aged (~50 weeks) WT vs. Mcpt6^−/−^ cells. As seen in Figure 2B, TSA caused a significant increase in the number of viable aged WT cells, whereas a significant reduction of the number of viable cells was seen in aged Mcpt6^−/−^ cells. These effects were reflected by corresponding changes in cell populations showing Annexin V positivity, i.e., TSA caused a decrease in the Annexin V positivity of aged WT cells, but caused an increase in Annexin V positivity in aged Mcpt6^−/−^ cells (Figure 2B). Hence, the absence of tryptase (Mcpt6) results in an increase in the sensitivity to HDAC inhibition, and this occurs in an age-dependent manner. This introduced the possibility that tryptase can have an impact on responses to cell stress caused by HDAC inhibition.

**Figure 3:**
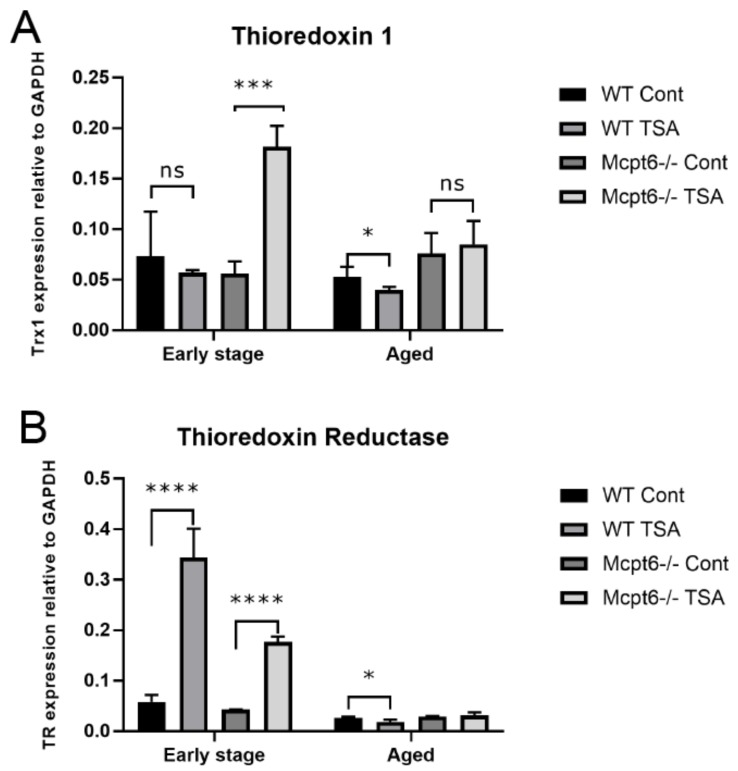
The absence of Mpt6 causes aberrant regulation of Thioredoxin 1 (Trx1) and Thioredoxin reductase (TR) in MCs exposed to TSA. WT and Mcpt6^−/−^ MCs (early stage and aged) were cultured ± TSA (see legend to Figure 2). Total RNA was isolated and was subjected to qPCR analysis for expression of (**A**) Trx1 and (**B**) TR. Note that aged WT MCs respond to TSA by downregulated expression of Trx1 and TR, whereas aged Mcpt6^−/−^ MCs were refractory. Expression of genes was evaluated relative to GAPDH. Data are presented as mean values ± SEM, pooled from three different experiments, analyzed with Sidaks multiple comparison test or by Two-way ANOVA. **** *p* ≤ 0.0001, *** *p* ≤ 0.001, * *p* < 0.01.

**Figure 4:**
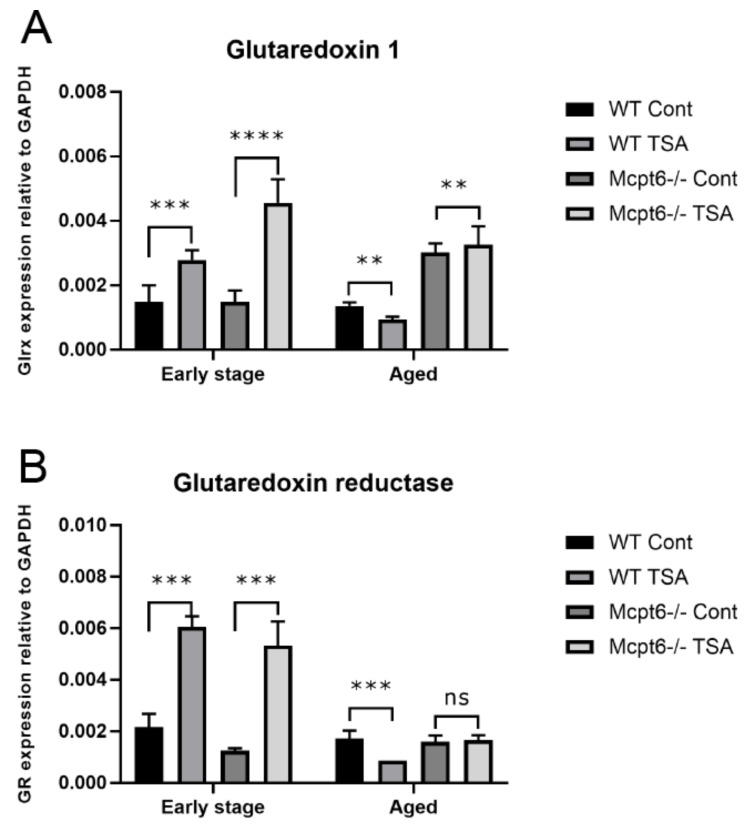
The absence of Mpt6 causes aberrant regulation of glutaredoxin 1 (Grx1) and glutathione reductase (GR) in MCs exposed to TSA. WT and Mcpt6^−/−^ MCs (early stage and aged) were cultured ± TSA (see legend to Figure 2). Total RNA was isolated and was subjected to qPCR analysis for expression of (**A**) Glutaredoxin 1 (Grx1) and (**B**) Glutaredoxin reductase (GR). Expression of genes was evaluated relative to GAPDH. Note that aged WT cells respond to TSA by downregulated expression of Trx1 and TR, whereas aged Mcpt6^−/−^ cells were either refractory (GR) or showed upregulated expression (Grx1) in response to TSA. Data are presented as mean values ± SEM, pooled from three different experiments, analyzed with Sidaks multiple comparison test or by Two-way ANOVA. *** *p* ≤ 0.001, ** *p* < 0.005. Cont, vehicle control.

To provide further insight into this we assessed whether tryptase could regulate the expression of genes operative in regulating oxidative stress. To this end we analyzed the effect of TSA on the expression of thioredoxin 1 (Trx1), thioredoxin reductase (TR), glutaredoxin 1 (Grx1), and glutathione reductase (GR) in WT vs. Mcpt6^−/−^ MCs. Trx1 is a redox active protein involved in defense against oxidative damage and TR is an enzyme having the function to reduce Trx1, thereby being essential for TR function. Grx1 and GR are both part of the glutathione antioxidative system [31,32]. When subjecting the early stage MCs to TSA, it was seen that WT cells were refractory in terms of Trx1 upregulation. In contrast, Mctp6^−/−^ cells responded to TSA with a robust upregulation of Trx1 (Figure 3A). Both WT and Mcpt6^−/−^ cells showed significant upregulation of TR expression in response to TSA (Figure 3B). In the aged cells, no significant effects of TSA on Trx1 or TR expression was seen in Mcpt6^−/−^ cells, whereas both Trx1 and TR were significantly downregulated in TSA-treated WT cells (Figure 3A,B). When examining the effects of TSA on Grx1 and GR, similar patterns as those seen for Trx1/TR were observed, i.e., aged WT cells responded to TSA by down-regulating these genes, whereas Mcpt6^−/−^ cells were either refractory (GR) or showed a slightly increased expression (Grx1). At the early stage, both WT and Mcpt6^−/−^ cells responded to TSA by up-regulating both genes (Figure 4A,B). Hence, these findings indicate that the absence of tryptase affects the ability of MCs to respond to cellular stress through induction of anti-oxidative mechanisms.

The findings above suggested that tryptase regulates cell numbers in response to TSA, which could potentially be linked to effects on proliferation. To address this, we investigated if tryptase can influence the expression of ribonucleotide reductase (RNR), a key enzyme in the generation of deoxyribonucleotides necessary for DNA synthesis. Notably, RNR is dependent on the Trx1/TR and Grx1/GR systems for activity, and the differential regulation of these enzymes in WT and Mcpt6^−/−^ MCs treated with TSA could thus reflect differential RNR function. RNR is composed of two subunits, R1 and R2. In the cell cycle S phase, canonical R2 is predominantly expressed but is replaced by p53R2 in quiescent cells (G_0_ phase). A high R2/p53R2 ratio is thus significative for proliferating cells, typically seen in transformed cells [31].

**Figure 5:**
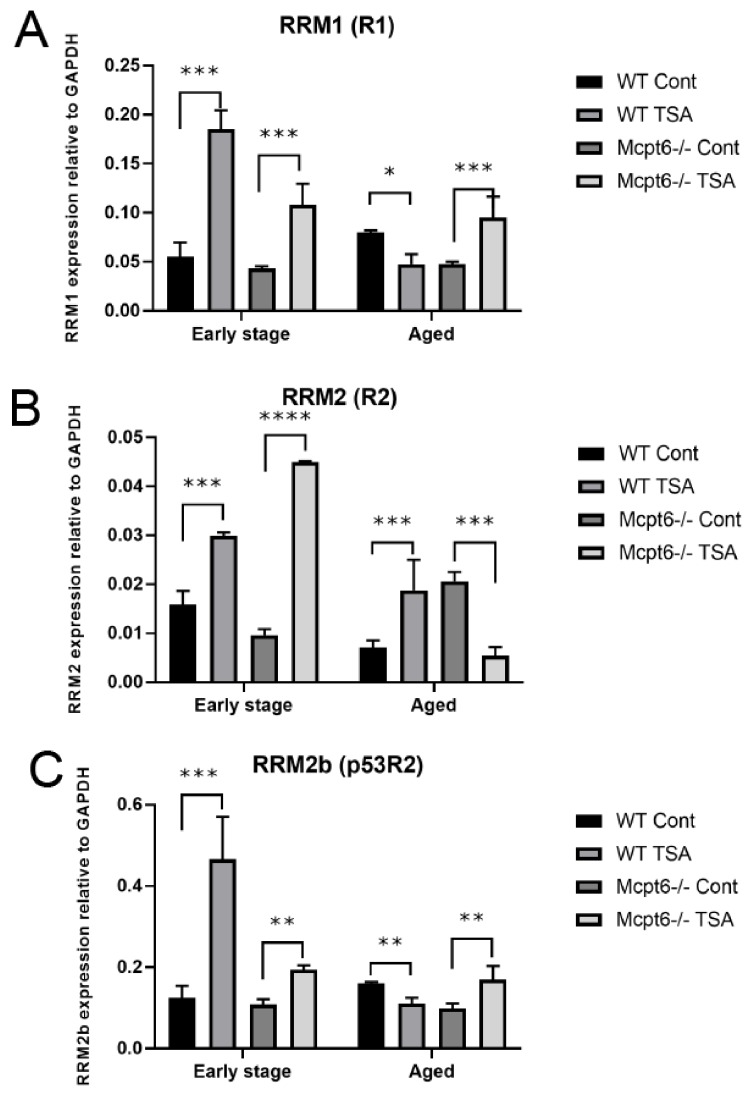
The absence of tryptase Mcpt6 causes dysregulated expression of the R1, R2, and p53R2 subunits of ribonucleotide reductase (RNR) in MCs exposed to TSA. WT and Mcpt6^−/−^ MCs (early stage and aged) were cultured ± TSA (see legend to Figure 2). Total RNA was isolated and was subjected to qPCR analysis for expression of (**A**) RNR R1 (RRM1/R1), (**B**) RNR R2 (RRM2/R2), and (**C**) RNR p53R2 (RRM2b/p53R2). Note that the effects of TSA on gene expression were similar in early stage WT vs. Mcpt6^−/−^ MCs, whereas contrasting effects of TSA on the regulation of RRM1, RRM2, and RRM2b were seen in aged WT vs. Mcpt6^−/−^ MCs. Expression of genes was evaluated relative to GAPDH. Data are presented as mean values ± SEM, pooled from three different experiments, analyzed with Sidaks multiple comparison test or by Two-way ANOVA. *** *p* ≤ 0.001, ** *p* < 0.005. Cont, vehicle control.

After exposure to TSA, early stage WT MCs responded by upregulation of all of these RNR subunits (Figure 5A–C). In the Mcpt6^−/−^ early stage cells, R1, R2, and p53R2 were also significantly induced by TSA treatment. However, it was noted that the TSA-induced upregulation of R1 and p53R2 was weaker than in WT cells, whereas the Mcpt6^−/−^ cells responded more vividly than WT cells in terms of R2 induction (Figure 5A–C).

In aged MCs, TSA caused a significant increase in R1 expression in Mcpt6^−/−^ cells but, in contrast, caused a reduction of the R1 expression in WT cells (Figure 5A). Similarly, p53R2 expression was reduced in TSA-treated WT cells but was significantly increased in corresponding Mcpt6^−/−^ MCs (Figure 5C). In contrast, R2 expression was stimulated in aged WT cells but was decreased to a major extent in cells lacking tryptase (Figure 5B). Hence, these findings suggest that tryptase has an impact on the regulation of the different RNR subunits in response to cellular stress caused by HDAC inhibition.

**Figure 6:**
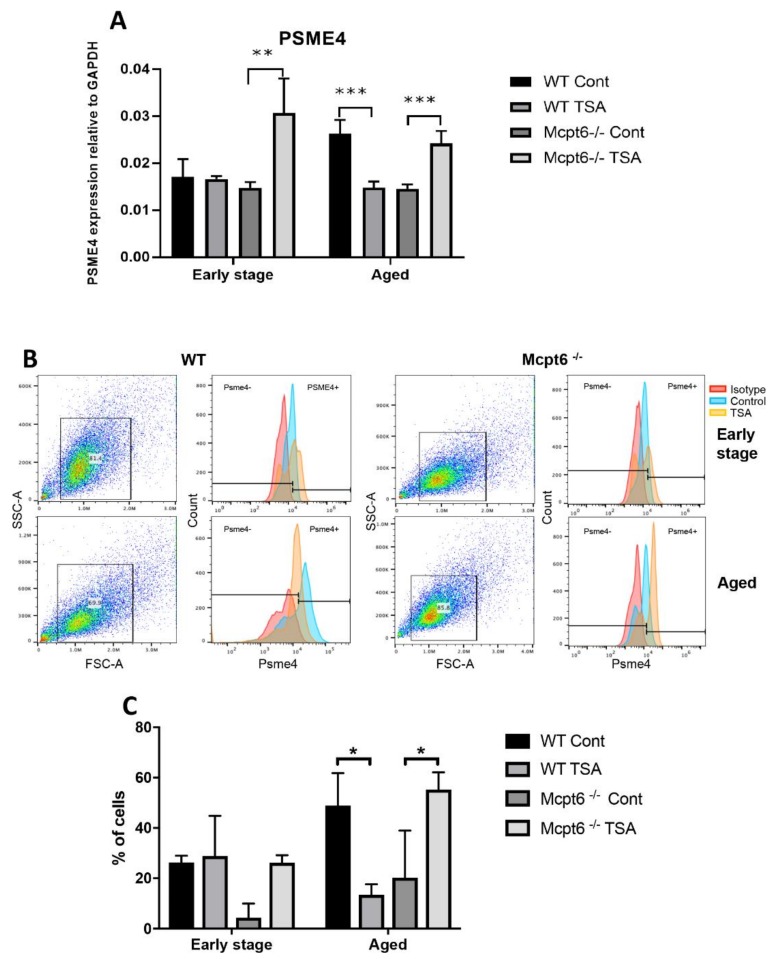
Tryptase Mcpt6 has an impact on the expression of Psme4/PA200 proteasomes in MCs. WT and Mcpt6^−/−^ MCs (early stage and aged) were cultured ± TSA (see legend to Figure 2). (**A**) Total RNA was isolated from WT and Mcpt6^−/−^ (early stage and aged) MCs, followed by qPCR analysis for expression of Psme4/PA200. Expression of genes was evaluated relative to GAPDH. Data are presented as mean values ± SEM, pooled from three independent experiments, analyzed with Sidaks multiple comparison test or by Two-way ANOVA. *** *p* ≤ 0.001. (**B**) Quantitative expression of Psme4/PA200 protein in WT and Mcpt6^−/−^ early stage and aged MCs, as determined by flow cytometry. Gates for Psme4/PA200 expression were placed in reference to isotype controls. Dot plots and histograms are representative of triplicates. (**C**) The data represent mean values from **B** ± SEM, * *p* < 0.01.

Altogether, these data suggest that tryptase has a major impact on the cellular responses caused by disturbed acetylation status of nuclear core histones. Thereby, we hypothesized that tryptase could play a role in regulation of mechanisms operative in maintaining a proper balance of acetylated histones. To date, there is very little insight into how this balance can be regulated. However, it has been shown that the PA200 proteasome (encoded by Psme4) can fulfill such a function in sperms [33], and also can have a similar role in transformed cell lines [34]. However, it is not known whether Psme4/PA200 is expressed in primary immune cells such as MCs and whether Psme4/PA200 can be linked to regulation of the histone acetylation in such cells. To approach this, we analyzed for the expression of Psme4/PA200 in MCs and asked whether Psme4/PA200 expression is affected by TSA and is influenced by the absence/presence of tryptase. As seen in Figure 6A, baseline Psme4/PA200 mRNA expression was seen in both the early stage and aged MCs. After treatment with TSA it was observed that Mcpt6^−/−^ cells responded by upregulation of Psme4/PA200 mRNA expression, whereas WT cells were either refractory (early stage cells) or showed downregulated expression (aged cells) (Figure 6A).

**Figure 7:**
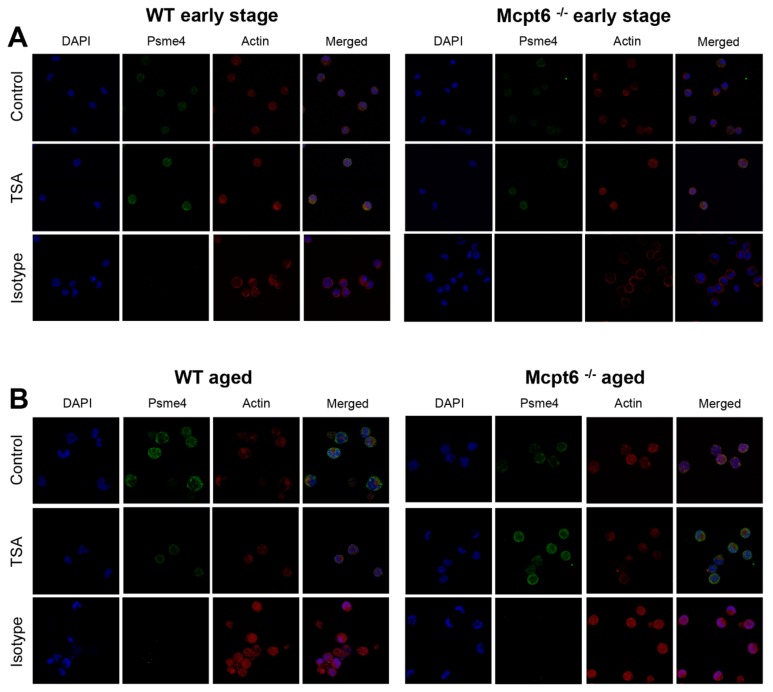
TSA causes upregulated expression of Psme4/PA200 in aged Mcpt6^−/−^ MCs but downregulated expression in aged WT MCs. Confocal microscopy analysis of early stage (**A**) and aged (**B**) WT and Mcpt6^−/−^ MCs that were either cultured alone (Control) or with TSA as indicated, followed by staining with DAPI (blue), Psme4/PA200 (green) and actin (red). The lower panels represent staining with the isotype control for the Psme4/PA200 antibody.

To provide support for these findings at the protein level we first performed flow cytometry analyses. These analyses confirmed that early stage WT MCs did not significantly upregulate the expression of Psme4/PA200 at the protein level in response to TSA, whereas a trend of upregulated Psme4/PA200 was seen in corresponding Mcpt6^−/−^ cells (Figure 6B,C). In agreement with the mRNA analysis, aged WT cells showed downregulated expression of Psme4/PA200 protein in response to TSA whereas, in contrast, TSA caused an upregulation of Psme4/PA200 protein in Mcpt6^−/−^ cells (Figure 6B,C). We also used confocal microscopy to visualize effects of TSA on Psme4/PA200. As shown in Figure 7, relatively weak baseline staining for Psme4/PA200 was seen in both WT and Mcpt6^−/−^ early stage MCs. After treatment with TSA, Psme4/PA200 staining was somewhat increased. In aged WT cells, TSA caused a profound decrease in Psme4/PA200 staining (Figure 7), in agreement with the qPCR and flow cytometry data. In contrast, TSA caused an increase in Psme4/PA200 staining in aged Mcpt6^−/−^ cells (Figure 7), which is in agreement with the qPCR and flow cytometry data. Hence, the absence of Mcpt6 is associated with aberrant expression of Psme4/PA200 proteasomes in response to cell stress caused by interference with histone deacetylation.

## 4. Discussion

MC tryptase has unique macromolecular properties, being built up as a tetramer where all of the active sites are facing a narrow central pore. Thereby, tryptase resembles the organization of proteasomes [35,36]. Due to this organization, tryptase is unique among serine proteases by being resistant to endogenous protease inhibitors. Tryptase activity is thus refractory to milieus rich in protease inhibitors such as plasma and cytosolic compartments. The regulation of tryptase activity is instead thought to occur through spontaneous, time-dependent dissociation of the tryptase tetramer, which leads to enzymatic destabilization [37,38]. Tryptase has previously been linked to the pathology of asthma (reviewed in [10]) and there are also reports implicating tryptase in a range of other settings, including tumor angiogenesis [39], melanoma cell proliferation [40], chronic obstructive pulmonary disease [41], arthritis [42], and acute experimental colitis [43].

The dogma has for a long time been that tryptase is exclusively localized to the MC secretory granules, and that the biological functions of tryptase are dependent on its release from MCs followed by effects on other cell types or on compounds residing in extracellular environment [17,19,38]. However, we recently challenged this notion by showing that tryptase, in addition to its location within the MC secretory granules, also can be found within the MC nucleus [25,28]. Moreover, we showed that tryptase can have an extensive functional impact on the MC itself, i.e., that tryptase can have cell-intrinsic functions in addition to its canonical cell-extrinsic functions [25,28]. When deciphering such cell-intrinsic functions, we noted that tryptase can regulate the proliferation of MCs [28], but the underlying mechanism was not revealed at that time. Moreover, we made the intriguing observation that tryptase has the ability to process the N-terminal ends of nuclear core histones, thereby removing epigenetic acetylation marks [25,28]. Another intriguing observation was that these effects were seen in aging MCs, but not in early stage MC cultures.

Based on these earlier observations, the present investigation sought to provide further insight into the cell-intrinsic properties of tryptase, with a particular focus on how tryptase affects processes related to regulation of histone acetylation, and if such effects are cell age-dependent. In support of such a hypothesis, previous studies have indicated that MCs are remarkably sensitive to HDAC inhibition [29], suggesting that an extensive accumulation of acetylated histones causes cellular stress to MCs.

Since our previous studies indicated that tryptase has the ability to regulate MC function in a manner dependent on histone acetylation [28], we reasoned that tryptase might have an impact on responses downstream of HDAC inhibition. Indeed, we show here that the absence of tryptase leads to increased sensitivity of MCs to toxicity induced by HDAC inhibition. Further, in agreement with our earlier observation that cell-intrinsic effects of tryptase are age-dependent, increased sensitivity of tryptase-deficient MCs to HDAC inhibition was only seen in MCs that had undergone extensive aging. The mechanism behind these findings is intriguing. A likely scenario, based on our previous work, is that the elevated sensitivity of Mcpt6^−/−^ MCs to HDAC inhibition might be due to differential levels of epigenetic histone acetylation marks in WT vs. Mcpt6^−/−^ MCs. Hence, this introduces the possibility that MC homeostasis in an aging context is dependent on adequate regulation of histone acetylation. This could be potentially be accomplished by regulation of enzymes that catalyze the acetylation/deacetylation of core histones. Alternatively, regulation of histone acetylation could be executed by proteolytic processing of core histones, whereby epigenetic histone marks are erased. The latter possibility has received very little attention, but there is emerging evidence from other cell types (sperms, transformed cell lines) that proteasomal degradation of core histones could serve such a function [33,34]. In this respect, proteasomes of the Psme4/PA200 type were recently shown to degrade nuclear histones and it was proposed that such degradation could serve to regulate the epigenetic profile of the cell [34]. Based on those observations we here hypothesized that the absence of tryptase could affect the levels of Psme4/PA200 proteasomes, thereby disturbing the appropriate turnover of core histones. In agreement with this notion, we show that the absence of tryptase leads to aberrant expression of Psme4/PA200. In agreement with effects of tryptase being cell-age dependent, aberrant expression of Psme4/PA200 was predominantly seen in aging MCs.

Based on these findings, we may propose that tryptase has an important role in maintaining an adequate status of acetylated histones, and thereby that toxic responses to HDAC inhibition are relatively modest in tryptase-expressing WT cells. In the absence of tryptase, the response to HDAC inhibition may thus become exaggerated and the cells might attempt to compensate this unbalance by increasing the expression of histone-degrading Psme4/PA200 proteasome units.

Overall, these findings suggest that tryptase-deficient MCs are more sensitive to cellular stress than are WT cells. As a further sign of this, we show that early stage tryptase-deficient MCs respond to HDAC inhibition by elevated upregulation of Trx1, an enzyme with a prominent role in the defense against oxidative stress. In addition, we noted that Trx1 expression in aged WT MCs was suppressed by HDAC inhibition, but was unaffected in tryptase-deficient cells. Similar patterns were seen also for TR, Grx1, and GR in aged cells, i.e., HDAC inhibition caused downregulation of the respective genes in WT cells whereas tryptase-deficient cells were largely refractory.

In addition to their roles in defense against oxidative stress, both Trx1/TR and Grx/GR are electron donors for RNR, an enzyme that has a key role in cellular proliferation by synthesis of deoxyribonucleotides to be incorporated into DNA. The absence of tryptase could thus, additionally, have an impact on RNR. In line with this, our data show that tryptase-deficiency is associated with differential regulation of the R2 subunit of RNR in response to cellular stress caused by HDAC inhibition. Since R2 (rather than p53R2) expression is typically associated with proliferative responses, it is conceivable that tryptase-dependent effects on R2 expression may account for our previous observation of elevated proliferative activity in MCs lacking tryptase [28].

It is important to emphasize that the findings presented here emanate from MCs that have aged in cell culture, and we are at present not able to ascertain that the observed effects of tryptase-deficiency are replicated in vivo in aging MC populations. However, we have previously shown that the absence of Mcpt6 causes an age-dependent expansion of MC populations in vivo, as well as increased size of MCs [28]. Hence, these findings suggest that tryptase indeed can have an impact on selected features of aging MCs in vivo, although further investigations will be required to specifically assess whether tryptase has an age-dependent impact on sensitivity of MCs to cellular stress in vivo.

In a broader perspective, we may also speculate that mechanisms such as those identified in this work may have an impact on aging in general. Specifically, we may speculate that maintaining of a proper balance of the histone acetylation status is a significant factor in the maintenance of cellular homeostasis, and that aging is associated with a significant challenge for the respective cell to prevent accumulation of epigenetic histone marks that otherwise might cause cellular damage. Possibly, tryptase may serve such a function in a MC context and it is also possible that similar mechanisms are operative in other cell types, although executed by enzymes other than tryptase. Accordingly, if this controlling mechanism is absent, as in tryptase-deficient MCs, aging cells may become more vulnerable to cellular stress as shown here for MCs. However, the validity of this hypothesis on a more general level remains to be investigated.
